# The treatment of wastewater containing pharmaceuticals in microcosm constructed wetlands: the occurrence of integrons (*int*1–2) and associated resistance genes (*sul*1–3, *qac*EΔ1)

**DOI:** 10.1007/s11356-017-9079-1

**Published:** 2017-05-10

**Authors:** Monika Nowrotek, Ewa Kotlarska, Aneta Łuczkiewicz, Ewa Felis, Adam Sochacki, Korneliusz Miksch

**Affiliations:** 1 Environmental Biotechnology Department, Silesian University of Technology, ul. Akademicka 2, 44-100 Gliwice, Poland; 2 Centre for Biotechnology, Silesian University of Technology, ul. B. Krzywoustego 8, 44-100 Gliwice, Poland; 3 Department of Genetics and Marine Biotechnology, Institute of Oceanology Polish Academy of Sciences, Powstanców Warszawy 55, 81-712 Sopot, Poland; 4 Department of Water and Wastewater Technology, Gdansk University of Technology, ul. Narutowicza 11/12, 80-233 Gdańsk, Poland; 5 Department of Applied Ecology, Faculty of Environmental Sciences, Czech University of Life Sciences Prague , Kamýcká 129, 165 00 Prague 6, Czech Republic

**Keywords:** Pharmaceuticals, Antibiotic resistance genes, Treatment wetlands, Transformation products

## Abstract

The aim of this study was to analyze the occurrence of sulfonamide resistance genes (*sul*1–3) and other genetic elements as antiseptic resistance gene (*qac*EΔ1) and class 1 and class 2 integrons (*int*1–2) in the upper layer of substrate and in the effluent of microcosm constructed wetlands (CWs) treating artificial wastewater containing diclofenac and sulfamethoxazole (SMX), which is a sulfonamide antibiotic. The bacteria in the substrate and in the effluents were equipped with the *sul*1–2, *int*1, and *qac*EΔ1 resistance determinants, which were introduced into the CW system during inoculation with activated sludge and with the soil attached to the rhizosphere of potted seedlings of *Phalaris arundinacea* ‘Picta’ roots (*int*1). By comparing the occurrence of the resistance determinants in the upper substrate layer and the effluent, it can be stated that they neither were lost nor emerged along the flow path. The implications of the presence of antibiotic resistance genes in the effluent may entail a risk of antibiotic resistance being spread in the receiving environment. Additionally, transformation products of SMX were determined. According to the obtained results, four (potential) SMX transformation products were identified. Two major metabolites of SMX, 2,3,5-trihydroxy-SMX and 3,5-dihydroxy-SMX, indicated that SMX may be partly oxidized during the treatment. The remaining two SMX transformation products (hydroxy-glutathionyl-SMX and glutathionyl-SMX) are conjugates with glutathione, which suggests the ability of CW bacterial community to degrade SMX and resist antimicrobial stress.

**Electronic supplementary material:**

The online version of this article (doi:10.1007/s11356-017-9079-1) contains supplementary material, which is available to authorized users.

## Introduction

Antimicrobial agents, together with antibiotic-resistant bacteria (ARB) and antibiotic resistance genes (ARGs), have been widely detected not only in the clinical settings but also in different environmental compartments (Aukidy et al. 2012; Ratola et al. 2012; Padhye et al. 2013; Hsu et al. 2014). Due to serious infections caused by multidrug-resistant bacteria, in 2014, the World Health Organization identified antimicrobial resistance as one of the most critical challenges and serious threats to the global public health (World Health Organization 2014). Thus, currently, both ARB and ARGs are regarded as emerging environmental contaminants, with possible implications for human health and ecological status of the environment (Sharma et al. 2016).

One of the major routes, by which antimicrobials, ARB, and ARGs are introduced into the natural ecosystem from the human settings, is wastewater discharge to the receiver. Although wastewater treatment plants significantly reduce the load of bacteria, the final effluents may contain ARB, sometimes even at higher concentrations than in the raw wastewater (Zhang et al. 2009; Novo and Manaia 2010; Luczkiewicz et al. 2010; Galvin et al. 2010; Mokracka et al. 2012; Pruden et al. 2012). To understand this phenomenon, particular attention has been given to the presence of selective pressure and mobile genetic elements (MGEs), which contribute to dissemination of resistance genes. Five classes of resistance integrons have been defined upon the sequences of integrase proteins (Cambray et al. 2010). Three classes of integrons are responsible for spreading the multidrug resistance, and among them, class 1 integrons have been commonly reported and suspected to serve as a potential gene pool for resistance genes in a variety of gram-negative and, to a lesser extent, gram-positive bacteria (Partridge et al. 2009). Majority of class 1 integrons are usually associated with genes encoding resistance to disinfectants (*qac*EΔ1) and sulfonamides (*sul*1), both widely used in the clinical practice (Toleman and Walsh 2011). *Sul*1 has been usually found on large conjugative plasmids and is associated with conserved segment (3-CS) of class 1 integrons (Radstrom et al. 1991; Antunes et al. 2007). To date, besides *sul*1, only two other genes encoding sulfonamide resistance (*sul*2–3) have been identified (Sköld 2001 *)*. The *sul*2 gene was considered to be mainly located on small non-conjugative plasmids (Radstrom et al. 1991), whereas s*ul*3 was reported in animals as well as human-liked sources (Luczkiewicz et al. 2013; Perreten and Boerlin 2003) and associated with non-classic class 1 integrons (Antunes et al. 2005). Most previously described class 2 integrons contain the same array of four gene cassettes, three antibiotic resistance gene cassettes (*dfrA1*, *sat*, and *aadA1*), conferring resistance to trimethoprim, streptothricin, and spectinomycin/streptomycin, respectively, and the *orfX* cassette of unknown function (Flores et al. 1990).

Sulfonamides belong to the important class of antimicrobial agents in the human and veterinary medicine, which were used even prior to the penicillin (Hoff et al. 2016). Among sulfonamides, sulfamethoxazole (SMX) is one of the most commonly prescribed antibiotics and is often combined with trimethoprim to treat a wide range of infections. Since SMX is excreted mainly in unchanged form, it has been also frequently found in the sewage systems. In the effluents from wastewater treatment plants (WWTPs), SMX was detected in the range from 0.004 to 9.46 μg/L (Trovó et al. 2009; Loos et al. 2010; Fatta-Kassinos et al. 2011; Luczkiewicz et al. 2013), while in the receiving waters up to 4 μg/L (Barnes et al. 2008).

SMX is a low-adsorptive and polar compound, with high mobility; thus, it is suspected to be removed from environment mainly due to the microbial activity (Banzhaf et al. 2012). In general, two bacterial groups might be responsible for the biodegradation of SMX: heterotrophic bacteria assimilating SMX-C (carbon deriving from SMX) and/or SMX-N (nitrogen deriving from SMX) and autotrophic nitrifying bacteria oxidizing the functional amino group on the aromatic ring of SMX under aerobic conditions (Müller et al. 2013). The removal efficiency in conventional activated sludge wastewater treatment systems was reported to be in the range 4–89% (Luo et al. 2014; Luczkiewicz et al. 2013; Miksch et al. 2016). The removal of SMX in constructed wetlands (CWs), which were the wastewater treatment systems used in the present study, depended on the type of the CW (Nowrotek et al. 2016). The three main types of CWs can be distinguished based on the flow direction and water position (relative to substrate): surface flow CWs (SF-CWs) and subsurface flow CWs with either horizontal (HF-CWs) or vertical flow (VF-CWs) (Fonder and Headley 2013). The most common type of VF-CWs are downflow (DF-CWs) intermittently fed systems with unsaturated bed (Nowrotek et al. 2016). The important feature of unsaturated DF-CWs is that they promote aerobic conditions allowing nitrification, which is in contrast to SF-CWs and HF-CW, which provide mostly anoxic conditions (Nowrotek et al. 2016). The elimination of SMX in CWs was relatively high in the systems, in which typically anoxic conditions prevail, in HF-CWs 73–97% (Hijosa-Valsero et al. 2011), in SF-CWs 59–92% (Hijosa-Valsero et al. 2011; Xian et al. 2010), and in DF-CWs with submerged (saturated) bed 63% in summer (but −74% in winter) (Rühmland et al. 2015). Low removal of SMX (24–31%) was observed in unsaturated DF-CWs (Nowrotek et al. 2016). The co-existence of various biotic and abiotic processes and the presence of various microenvironments in the CWs allow to treat these systems as an interesting alternative to conventional treatment methods regarding the removal of standard wastewater pollutants but also organic micropollutants including pharmaceuticals (Ávila et al. 2010; Li et al. 2014; Zhang et al. 2012; Matamoros et al. 2007). So far, there have been, however, only few reports related to the presence of ARB and ARGs in CWs (Huang et al. 2015; Chen et al. 2015; Liu et al. 2014; Nõlvak et al. 2013; Liu et al. 2013; Sharma et al. 2016). Noteworthy, the temporal changes of the occurrence of ARGs in the CW substrate have not been assessed, which was a motivation for the present study.

The aim of this study was to analyze the occurrence of *sul*1–3, *qac*EΔ1, and class 1 and class 2 integrons (*int*1–2) genes in the substrate and effluents of microcosm unsaturated DF-CWs fed with synthetic wastewater containing pharmaceuticals (SMX and diclofenac (DCF)) taking into account the effect of the inoculating materials and the effect of plants. Another objective of this study was to identify the transformation products of SMX (TPs-SMX) in the effluents of the studied CWs. The potential relation between the resistance determinants, the presence of SMX, TPs-SMX, and the plants will help to better understand the adaptive evolution of microorganisms in the environment subjected to a strong selective pressure.

## Materials and methods

### Experimental system

The microcosm experimental system (photo presented in Fig. S1, Supporting Information (SI)) used in this experiment was constructed and operated to mimic the behavior of DF-CWs and was treating synthetic high-strength municipal wastewater containing pharmaceutical compounds (PhCs): SMX and DCF. These compounds were added in a mixture at a concentration of 5 mg/L each, dissolved in high-strength synthetic municipal wastewater prepared in tap water according to the protocol of Nowrotek et al. (2016). The composition of the synthetic wastewater is presented in Table S1 (SI). The characteristics of the experimental system were described in detail by Nowrotek et al. (2016); however, the most relevant features of the system will be recalled here. The experimental system consisted of 24 columns (diameter 0.2 m) filled up to 0.7 m with filtering media (bottom layer: gravel; main layer: quartz sand; and uppermost layer (5 cm): a mixture of sand and organic soil, which originated from the seedlings of the plants). Twelve out of 24 columns were planted with reed canary grass ‘Picta’ (*Phalaris arundinacea* L. var. *picta* L.). The sand mixed with the soil originating from the pots of the seedlings of plants was also added as the upper layer in the unplanted columns in order to ensure comparable substrate conditions in all the columns. Finally, four types of columns (in six replicates) were used. The following symbols were used to denote the columns: planted columns fed with PhCs (PhCs-P), unplanted columns fed with PhCs (PhCs-U), and their respective control columns, planted or unplanted, fed only with synthetic wastewater (noPhCs-P and noPhCs-U), respectively.

During the experiment, all the columns were fed intermittently in the feeding/resting regime of 5 days (feeding from Monday to Friday)/2 days (resting on Saturday and Sunday). On the feeding day, each column was fed with 1 L of the influent in a single pulse (Nowrotek et al. 2016). This corresponds to hydraulic loading rate of 0.032 m^3^ of the influent per square meter of the bed top surface in a single pulse (which equals 32 mm/pulse as commonly expressed). All the columns were operated as unsaturated DF-CWs (free-draining beds). The columns were fed manually using a graduated pitcher from the top and the wastewater percolated by gravity to the outlet situated at the bottom of the columns. The settings of the lighting system, which was used to provide grow light for the plants growing in the CW system, were reported in Nowrotek et al. (2016).

### Timeline of the experiment and sampling

The experiment started with the pre-incubation period, which lasted 217 days. During the pre-incubation period, all the columns were fed with the synthetic wastewater containing no PhCs. In the initial 5 days of the pre-incubation period, all the columns were inoculated with the activated sludge. To this end, 1 L of activated sludge, which contained 3.5–4.0 g mixed liquor suspended solids/L, was spread on the top of each column. The activated sludge was obtained from the local, municipal WWTP. At the beginning of the pre-incubation period, the following samples were taken: the activated sludge used for column inoculation, the soil attached to the roots and rhizomes of the potted seedlings of reed canary grass ‘Picta’, and the tap water used to prepare the feed. The samples of these media were taken before they were introduced into the columns.

**Fig. 1:**
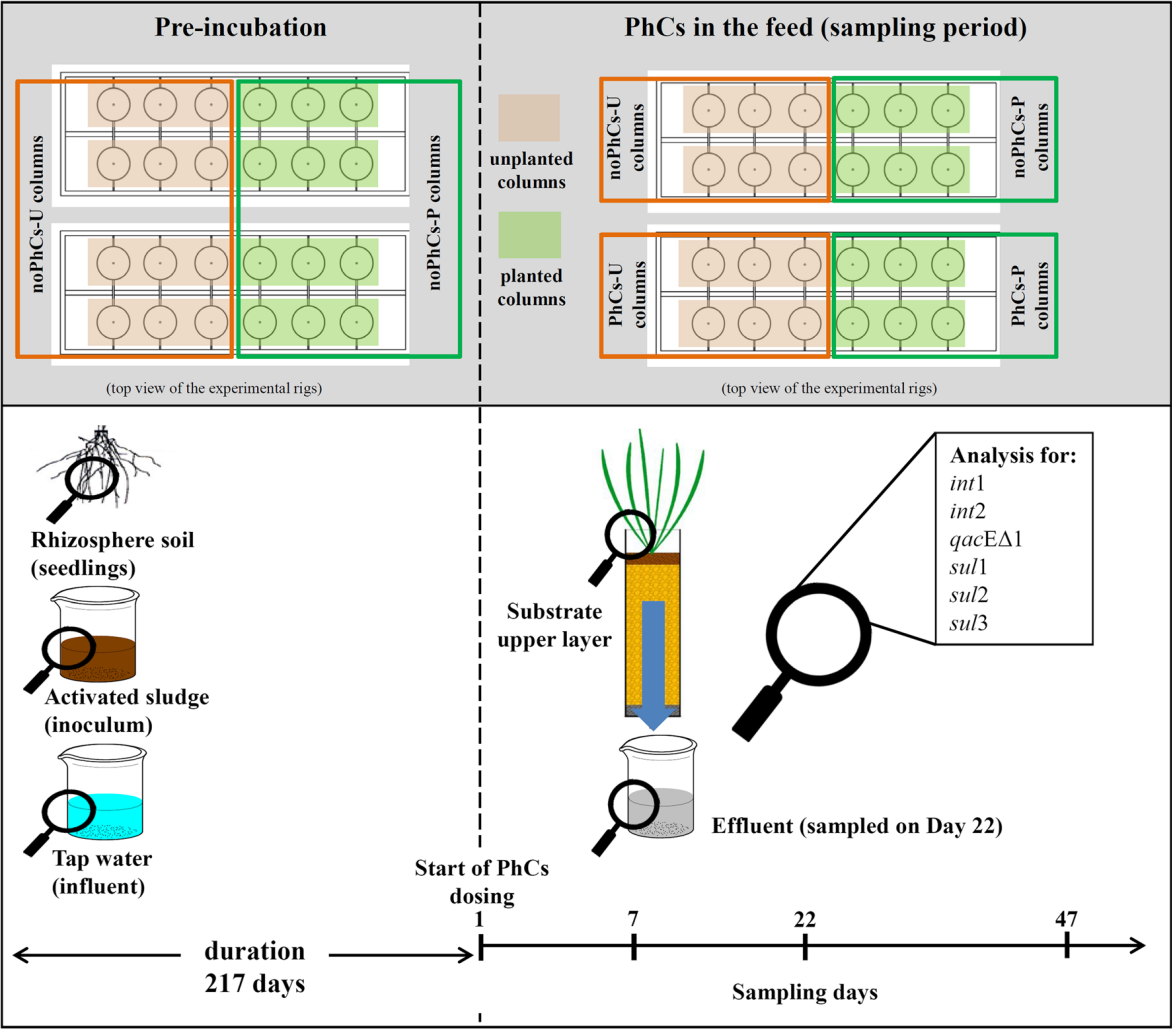
Experiment and sampling timeline and sampling spots

The main part of the experiment started after the pre-incubation period and lasted next 86 days. During that part, the PhCs-P and PhCs-U columns were fed with wastewater containing PhCs, while the remaining columns were still fed only with artificial wastewater (noPhCs-P and noPhCs-U). Noteworthy, as mentioned in “Experimental system,” the influent, which was used to feed the PhCs-P and PhCs-U columns, contained also DCF. During the main part of the experiment, the following samples were taken from each column: the upper layer of substrate (10-cm core sample of the uppermost layer of the substrate) on days 7, 22, and 47, as well as the samples of column effluents on day 22. The samples were taken from the rhizosphere zone, because in the case of the DF-CWs, about 95% of the bacterial activity was concentrated in the first 10 cm of the sandy substrate filter (Tietz et al. 2007). In the present study, the intention of the authors was to analyze the qualitative variability of resistance genes in the zone where the content of the microbial biomass is the highest. The timeline of the experiment and sampling along with the overview of sampling spots is presented in Fig. 1.

### DNA extraction

DNA was extracted from the following samples: soil attached to the roots and rhizomes of *P. arundinacea* ‘Picta’ seedlings prior to being planted in the columns, from the activated sludge used for the columns inoculation, from the tap water used for preparation of synthetic wastewater, the upper layer of substrate of all the columns at various stages of the experiments, and the samples of the effluents (Fig. 1). All the samples (substrate and effluent) from which DNA was subsequently extracted were frozen at −20 °C and kept for future analyses together with samples needed for the background study (taken during the pre-incubation period). For isolation of the total bacterial DNA, mechanical method was used with glass beads of different diameters. The soil and substrate samples (5 g DW) and activated sludge sample (1 g DW) before isolation were shaken for 16 h in 10 mL of 1× phosphate-buffered saline (PBS, pH 7.4) using the shaker. This phase was used to remove potential PCR inhibitors and to release the bacteria from the soil matrix. After shaking and following centrifugation, the supernatant was collected repeatedly from the resulting slurry to obtain 0.2 g of the precipitate. The samples of the effluents as well as the tap water used for preparation of synthetic municipal wastewater were filtered using isopore polycarbonate 0.22-μm filters (Merck). Then, the filters were dissected into pieces and processed as the soil samples in order to isolate the total DNA from the bacterial cells attached to the filters. The prepared samples were rinsed three times with 1× PBS. Then, the samples were centrifuged, and extraction buffer was added (100 mM Tris-HCl, 100 mM EDTA, 1.5 M NaCl, pH = 8). One hundred fifty milligrams of bead beating with different diameters was added to all samples (1.25–1.55, 0.4–0.6 mm; Roth, Germany). The material was suspended in a solution by intensive vortexing. The samples were incubated 20 min with shaking at 1400 rpm, using a thermomixer (Eppendorf), and after this, 200 μL 10% SDS was added. After 30 min of incubation at 65 °C, the samples were centrifuged twice at 13,000 rpm and placed on column spin filters (A&A Biotechnology). The filter was washed twice with 70% ethanol solution (A&A Biotechnology), and the DNA attached to the filter was eluted with sterile distilled water. The DNA was stored at −20 °C until PCR amplification. The quality of the DNA was checked using agarose electrophoresis and spectrophotometer. The presence of bacterial DNA was confirmed using PCR with primers F27 and 1492R (Lane 1991).

### Detection of selected resistance determinants

PCR assays were developed for the specific detection of the genes *sul*1–3, *int*1, *int*2, and *qac*EΔ1. In all the experiments, DNA from *Escherichia coli* DH5α served as a negative control, and DNA from strains from the previous work was used as a positive control (Luczkiewicz et al. 2013; Kotlarska et al. 2015). All primers used in this study are shown in Table S2 (SI) and were based on the works of Goldstein et al. (2001), Grape et al. (2003), Kraft et al. (1986), Perreten and Boerlin (2003), Stokes and Hall (1989), and Toleman et al. (2006).

PCR was performed in a volume of 10 μL which contained appropriate primers (5 pmol/μL), the dNTPs 0.2 μL (20 pmol/μL, Promega), 2 μL of 10× of reaction Taq buffer MgCl_2_ free (Promega), 0.1 μL of Taq DNA Go Flexi polymerase (1.5 U, Promega), and 0.5 μL of the extracted DNA. The cycling program consisted of an initial denaturation (94 °C for 9 min), followed by 30 cycles of denaturation (94 °C for 30 s), annealing (30 s at temperature indicated in Table S2 (SI)), and extension (72 °C for 10 min), as it was described in Luczkiewicz et al. (2013) and Kotlarska et al. (2015). PCR products were analyzed by electrophoresis on a 1% agarose gel in 1× TAE buffer (Sambrook and Russell 2001) and stained with ethidium bromide (0.5 μg/mL), visualized under UV light, and documented using Vilber Lourmat image acquisition system. The size of the PCR products was compared with 1 kb DNA ladder (Thermo Scientific) in Bio1D software (Vilber Lourmat, Marne-la-Vallée, France).

### Chemical analysis

Standard wastewater parameters such as chemical oxygen demand (COD), ammonium nitrogen (N-NH_4_), ortophosphate phosporus (P-PO_4_), nitrate nitrogen (N-NO_3_), total organic carbon (TOC), and total nitrogen (TN) were analyzed according to the protocol of Nowrotek et al. (2016).

High-performance liquid chromatography method was used to determine the SMX concentrations in the influent and effluents between days 1 and 86 of the experiment. The analytical protocol for this method was reported in Nowrotek et al. (2016). The limit of quantification (LOD) of SMX was equal to 0.2 mg/L.

The effluent samples (taken on day 22) from the PhCs-P columns were analyzed by LC-MS/MS for the presence of TPs-SMX. The effluents from the corresponding control columns were taken as background samples during the analysis of the transformation products. The identification of the TPs-SMX was performed using ultra-performance liquid chromatography system (UltiMate 3000 RS; Dionex) coupled with a tandem mass spectrometer (AB SCIEX 4000 Q TRAP) equipped with an electrospray ionization source (ESI). In both cases, the chromatographic separation was performed using C18 Hypersil™ Gold column (250 mm × 4.6 mm; pore size 5 μm) (Thermo Scientific, Polygen, Poland) at an isocratic operation mode. The flow rate of the mobile phase through the chromatographic column was equal to 1.0 mL/min. The mobile phase was composed of a mixture of acetonitrile and phosphate buffer (pH = 5.7; 40/60; *v*/*v*) and 0.1% formic acid (40/60; *v*/*v*). The tandem mass spectrometry (with electrospray ionization) was performed in a positive ion mode. Crucial MS-MS detector parameters such as ionization voltage (IS), collision-assisted dissociation (CAD) gas, source temperature (TEM), sheath gas (GS1), desolvation gas (GS2), and curtain gas (CUR) were optimized by flow injection analysis (FIA) in order to obtain better ionization of the pattern compound. These parameters were as follows: TEM = 500 °C, IS = 4000 V, CAD = medium, CUR = 15 psi, GS1 = 35 psi, and GS2 = 35 psi. The identification of TPs-SMX was performed by means of LightSight® software (AB Sciex) using predicted multiple reaction monitoring (pMRM) mode.

### Statistical analysis

Statistical testing was performed using the STATISTICA 12 software (Stat Soft Inc. 2013). The results concerning the removal of SMX, TOC, and N-NO_3_ have been analyzed according to the procedure discussed in Nowrotek et al. (2016). The chi-squared and Fisher’s exact tests were performed to determine the relationship between the presence of analyzed genes, concentration of SMX, and the presence of plants in columns. The hypothesis was rejected at a *p* value ≥0.05.

## Results and discussion

### Performance of the experimental system

The performance of the experimental system in the discussed period was described in detail by Nowrotek et al. (2016). The removal of DCF is not going to be mentioned in this article, because its effect on the antimicrobial resistance in bacteria was beyond the scope of this study. The SMX removal efficiency was 24.22 ± 14.66% (average ± standard deviation) and 30.86 ± 14.57% in PhCs-P and PhCs-U columns, respectively, and no statistically significant differences were found between these columns. Hence, it was concluded that the presence of plants had no effect on the removal process. Additionally, the presence of PhCs in the feed did not affect the removal of COD, N-NH_4_, TN, and P-PO_4_ (Nowrotek et al. 2016) but affected significantly the removal of TOC and N-NO_3_. The effluent TOC concentration was higher in the columns fed with the influent containing PhCs than in the control columns, which could have resulted from the presence of the PhCs and their TPs in the effluent from the PhCs-P and PhCs-U columns. In the case of N-NO_3_, its concentration was higher (by less than 20 mg N-NO_3_/L) in the no-PhCs columns (Nowrotek et al. 2016). The underlying mechanism was not studied, but it was hypothesized that the PhCs could have impaired nitrification. However, the biodiversity of ammonia-oxidizing bacteria throughout the experiment did not change significantly and was not affected by the PhCs (Nowrotek et al. 2015).

### Transformation of SMX in planted columns

**Fig. 2:**
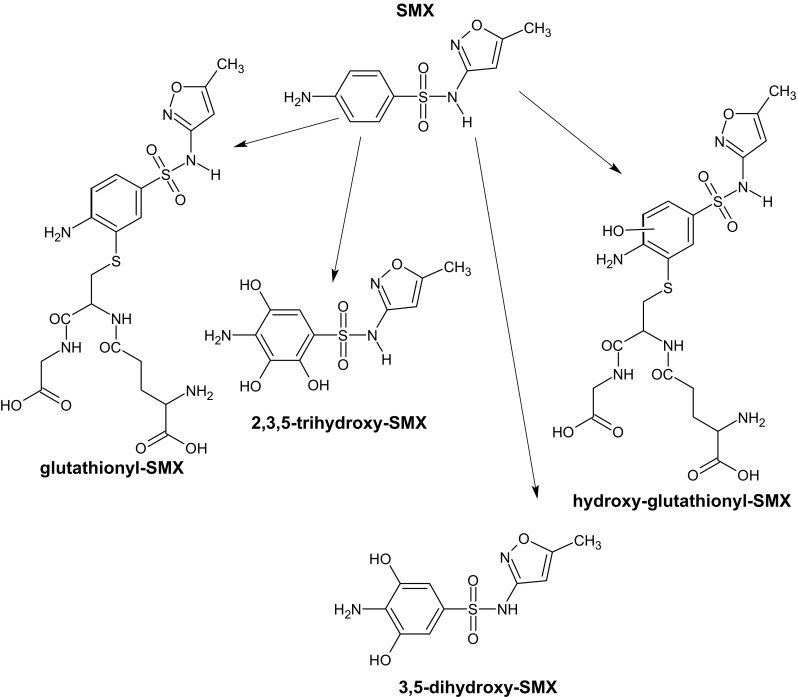
Candidate transformation products of SMX during its degradation in the PhCs-P columns

Several studies have clearly indicated that sulfonamides are mobile in saturated porous media and can be removed in CWs mainly by means of biodegradation and/or plant uptake (Conkle et al. 2008; Chen and Zhang 2013; Banzhaf et al. 2012), with sorption onto soil or wetland media being of lesser importance. In this study, the presence of TPs-SMX was analyzed in the effluents from the PhCs-P columns on day 7 of the experiment (Fig. 1). The structures of four potential TPs-SMX were proposed (Fig. 2).

The two major TP-SMXs were trihydroxy-SMX and dihydroxy-SMX (probably 2,3,5-trihydroxy-SMX and 3,5-dihydroxy-SMX, but the placement of the hydroxyl substituents in the benzene ring was not unambiguously confirmed). This indicated that SMX may be partly oxidized during the treatment in unsaturated CWs. In the case of activated sludge, 3-amino-5-methyl-isocazole was the main stable SMX biodegradation product (Müller et al. 2013). Also, in samples from municipal effluent wastewater (activated sludge treatment plant), SMX hydroxylamine was detected as a major product in aerobic biodegradation (Poirier-Larabie et al. 2016).

The two other transformation products hydroxy-glutathionyl-SMX and glutathionyl-SMX are conjugates with glutathione (GSH), which is a marker of an oxidative stress in bacteria and plants. In plants, GSH is involved in a plethora of cellular processes, including defense against reactive oxygen species (Foyer and Noctor 2005; Mullineaux and Rausch 2005), sequestration of heavy metals (Cobbett and Goldsbrough 2002; Freeman et al. 2004), and detoxification of xenobiotics (Dixon et al. 1998). However, in the plants, there is lack of an excretory pathway; thus, the metabolites must be stored in the plant tissues (Bartha et al. 2014). In prokaryotic cells, GSH is found chiefly in gram-negative bacteria including *E. coli*. In most gram-positive bacteria, with the exception of some *Streptococcus* and *Enterococcus* species, GSH was not found (Fahey et al. 1975; Newton et al. 1996). The presence of GSH conjugates with SMX can attest to the adverse effect of this substance on microorganisms or plants or both.

### Occurrence of resistance determinants in the substrate and effluents

The presence of TPs-SMX (Fig. 2) in the effluents indirectly confirmed that SMX underwent biotransformation in the studied CW system. Thus, the presence of *sul*1–3 together with integrons was suspected among the CW bacterial community. Since sulfonamides are synthetic antimicrobials, only acquired resistance is suspected in bacteria. Therefore, in this study, it was crucial to determine the possible source of resistance determinants. According to the obtained results, none of the tested resistance determinants were found in the tap water, only *int*1 was present in the soil sampled before the experiment, whereas *sul*1–2, *qac*EΔ1, and *int*1 were detected in the samples of activated sludge. The activated sludge has been already reported as a niche rich in *sul*1–3 and integrons (e.g., Tennstedt et al. 2003). Those resistance determinants were also detected in the activated sludge samples obtained from the same WWTP and used in the previous study (Luczkiewicz et al. 2013; Gnida et al. 2014). In wastewater processes, class 1 integrons are suggested to serve as an important reservoir of resistance genes, mainly those encoding resistance to disinfectants (*qac*EΔ1) and sulfonamides (*sul*1) (Enne et al. 2001; Huovinen et al. 1995; Huovinen 2001; Sköld 2001). The presence of integrons is also regarded as an example of adaptive evolution of bacterial community to the environmental stresses, such as presence of subinhibitory concentrations of antimicrobial agents, heavy metals, varying amounts of oxygen, and others (Aminov 2011). Since in this study the *int*1, *sul*1–3, and *qac*EΔ were introduced to each column with activated sludge and the soil, the presence of those resistance determinants was studied in each upper substrate layer and in the effluents of PhCs-P and PhCs-U columns. Summary of the results obtained in the course of the experiment is given in Table 1, while the detailed results for each column replicate are given in Table S3 (SI).

**Table 1:** The occurrence of the analyzed genes (*sul*1–3, *int*1–2, and *qac*EΔ1) at various stages of the CW experiment (number of replicates in which the presence of a given determinant was detected)

Column	Resistance determinants	PhCs in the feed		
		Day 7	Day 22	Day 47
Upper layer of substrate (*n* out of 6 replicates)				
PhCs-P	*sul*1	2	6	5
	*sul*2	4	5	1
	*sul*3	0	0	0
	*int*1	2	6	1
	*int*2	0	0	0
	*qac*EΔ1	0	6	0
noPhCs-P	*sul*1	1	2	2
	*sul*2	4	3	0
	*sul*3	0	0	0
	*int*1	1	2	0
	*int*2	0	0	0
	*qac*EΔ1	0	0	0
PhCs-U	*sul*1	1	5	3
	*sul*2	2	4	0
	*sul*3	0	0	0
	*int*1	2	5	1
	*int*2	0	0	0
	*qac*EΔ1	0	5	0
noPhCs-U	*sul*1	0	1	0
	*sul*2	1	1	0
	*sul*3	0	0	0
	*int*1	1	0	0
	*int*2	0	0	0
	*qac*EΔ1	0	0	0
CW effluents (*n* out of 6 replicates)				
PhCs-P	*sul*1	nt.	6	nt.
	*sul*2	nt.	6	nt.
	*sul*3	nt.	0	nt.
	*int*1	nt.	6	nt.
	*int*2	nt.	0	nt.
	*qac*EΔ1	nt.	6	nt.
PhCs-U	*sul*1	nt.	5	nt.
	*sul*2	nt.	5	nt.
	*sul*3	nt.	0	nt.
	*int*1	nt.	5	nt.
	*int*2	nt.	0	nt.
	*qac*EΔ1	nt.	5	nt.

*nt*. not tested

#### Occurrence of *sul*1–3 genes in substrate and effluents

The 217-day pre-incubation period, which is regarded as a relatively long-term absence of selective pressure of antimicrobials, could have caused the loss of resistance determinants among the bacterial cells introduced into the columns with activated sludge and the soil. Since most antibiotic resistance mechanisms are associated with fitness costs, typically observed as a reduction of bacterial growth rate, it was suspected that in the absence of selective pressure, the sensitive bacteria would have replaced the resistant ones (Andersson and Hughes 2010). But as it can be seen in Table 1, most of the resistance determinants (with the exception of *qac*EΔ1) that had been detected in the inoculum were, however, still present in the substrate on day 7, and only minor differences between the CWs fed with PhCs and their control counterparts were observed. It can be hypothesized that the stability of bacterial resistance can be promoted by the presence of several potentially toxic metals in the feed (Table S1). The co-selection of heavy metal and antimicrobial resistance has been already proved by several studies (e.g., Baker-Austin et al. 2006; Seiler and Berendonk 2012). Some studies, investigating co-selection in the environment, frequently showed the correlation of increased heavy metal concentrations with increased phenotypic or genotypic antibiotic resistance (Berg et al. 2005; Berg et al. 2010; Knapp et al. 2011). The non-correspondence contaminants, such as Cu, Ni, Zn, and Pb, were implicated in the abundance of *sul*1–3 (especially when the genes specifying resistant phenotypes are located together on the same genetic element such as integron) and *int*1 genes (Lu et al. 2015). All of these metals were added to the prepared municipal wastewater during the experiment (Table S1, SI). In this study, the concentration of heavy metals present in the feed was not determined, but their effect on the ARGs cannot be excluded. It is also suggested that dissemination of antibiotic resistance can be supported by the presence of heavy metals, particularly in the niches, where the antibiotic concentration is very low (Lu et al. 2015). Moreover, the phytostabilization of some heavy metals was observed to be mediated by *P. arundinacea* (reed canary grass) (Polechońska and Klink 2014). Thus, in this study, it can by hypothesized that the presence of plants in the columns and heavy metals in synthetic wastewater, which was fed into the columns, is conducive to emergence of sulfonamide resistance genes (*sul*1 and sul2) and *int*1 in the rhizosphere zones. It can also explain the presence of *sul*1–2 and *int*1 genes in noPhCs-P columns. Whereas, the absence of *sul*1–2 and *int*1 genes in noPhCs-U columns partly corroborates this hypothesis. Therefore, there is inevitability for further research in this subject. However, besides the fitness costs, the resistance determinants can be also lost by the leaching of the resistant bacteria from the upper substrate layer (or rhizosphere in the case of planted columns) or even out of the CW columns.

Some studies have shown that the number of *sul*1–2 genes increased with the depth of sampling (Liu et al. 2014). However, the factors involved in the maintenance and distribution of resistance determinants within natural communities to date are largely unknown. Interestingly, in the soil samples collected on day 7, predominantly the *sul*2 gene was detected (Table 1). The *sul*2 gene was found in equal number (in four replicates) of planted columns regardless of the feed, while in the unplanted columns, *sul*2 was detected only in two columns fed with PhCs (PhCs-U) and in one control column (noPhCs-U). The *sul*1 gene was found less frequently than *sul*2 (Table 1). It should be noted that the correlation between the presence of *sul*1, *sul*2, *int*1, and *qac*EΔ1 genes and the presence of plants on day 7 (225 days of experiment) was found to be significant (*p* < 0.05) only in the case of the *sul*2 genes.

On day 22, both *sul*1 and *sul*2 genes were detected together with *int*1 and *qac*EΔ1 in almost all soil samples collected from the columns fed with PhCs, with invariably higher number of replicates containing specific resistance determinants in the case of PhCs-P columns. In the case of the control columns (noPhCs-P and noPhCs-U), the resistance in general remained on the similar level as on day 7. On day 47, mainly *sul*1 was detected in the PhCs-P and the PhCs-U columns and also in the noPhCs-P columns, while in the PhCs-U columns, none of tested resistance determinants was found. On day 22, significant relationship was observed between the presence of the *sul*1, *sul*2, and *int*1 genes and the presence of the plants in the columns, and on day 47, no significant relationships between the presence of resistance determinants and plants were observed.

The results obtained in the experiment suggest that bacterial community in rhizosphere zone of studied CW system remained equipped with the tested resistance determinants originating from the inoculum, despite the lengthy absence of antimicrobial pressure in the pre-incubation period. Additionally, the presence of *P. arundinacea* ‘Picta’ was to some extent positively correlated with the presence of tested determinants, but the differences in the SMX removal in these columns were insignificant (Nowrotek et al. 2016). Additionally, in the noPhCs-P columns, the *sul*1–2 and *int*1 genes were also detected in higher number of replicates and for a longer period compared to the noPhCs-U columns, despite the lack of antimicrobial agents in the feed. It was suggested, e.g., by Brown (1961) or by Harris and Woodbine (1967), that the survival of rhizospheric bacteria can be also associated with the enhanced resistance to several environment stressors. The potential ecological significance of this phenomenon was, however, under dispute, since studies mentioned above were based on the culture-dependent techniques. Nevertheless, even now, microbial/plant interactions in terms of antimicrobial stress are not clear and require essential knowledge of several factors, such as detailed characterization of bacterial community and plant physiology as well as the affinity of antimicrobial agents to adsorb and to undergo biodegradation, photodegradation, or plant uptake. Also, other stress factors, in case of this study, mainly heavy metals present in the feed, should be considered.

#### Occurrence of *int*1 genes in substrate and effluents

The results obtained in the experiment period, especially on day 22, showed the common presence of *int*1 in PhCs-P, PhCs-U, and noPhCs-P columns. The *int*1 gene was also detected on day 7 in all the types of columns (but in only one or two replicates out of six) and day 47 in single PhCs-P and PhCs-U columns. Class 1 integrons are suspected to be widely involved in the spread of antibiotic resistance among gram-negative bacteria, and the correlation between the abundance of *int*1 and the abundance of antibiotic and heavy metals resistance genes has been already noted (Wright et al. 2008).

In this study, *int*1 was simultaneously detected with *sul*1 (and to a lesser extent with *qac*EΔ1), mainly in the columns fed with PhCs-containing wastewater, which to some extent reflects the general response of the bacterial community to imposed selection pressure. On the other hand, the presence of resistance determinants changed with time in all the types of columns. Liu et al. (2014) showed higher abundance of *sul*1 and *sul*2 genes in the lower part of DF-CWs. Therefore, it can be assumed that bacteria with resistance determinants can be transferred to the lower part of the columns. In this study, such bacterial washout in the columns was confirmed by the results obtained for the effluents, which were collected from PhCs-P and PhCs-U columns on day 22. All the effluents contained *sul*1–2, *int*1, and *qac*EΔ1.

Thus, future detailed study is needed to analyze the bacterial response to antimicrobial stress in terms of biofilm formation and migration through the substrate profile in a column. It is particularly important due to possible groundwater contamination. Also, the resistance determinants should be quantified and related to the possible changes of bacterial community, apart from the upper substrate layer also at other depths of the substrate. It would be also advisable to evaluate simultaneously the presence of possible stressors, besides local concentration of antimicrobial agents also bioavailability of heavy metals and concentration of oxygen and/or redox potential. Additionally, the antibacterial activity of TPs-SMX may play a role in shaping the bacterial community in CWs; therefore, this aspect should be also taken into account (Majewsky et al. 2014).

## Conclusions and outlook

In this study, it was observed that the quality of the sulfonamide resistance genes changed during the exposure to the wastewater containing SMX. Moreover, this study indicated that the detected resistance determinants were only those which were introduced to the CW system with activated sludge (*sul*1–2, *int*1, and *qac*EΔ1) and with the soil attached to *P. arundinacea* ‘Picta’ roots and rhizomes (*int*1). The obtained results indicate that even extended lack of antimicrobial pressure did not cause the total loss of resistance ability. It can be suggested that the bacterial fitness costs could be mitigated during the entire experiment by the presence of heavy metals in artificial wastewater and to some extent by the presence of plants. Furthermore, the results of this experiment may indicate the need for further research on to the occurrence of sulfonamide resistance genes in CWs with special focus on the effect plants and heavy metals present in the wastewater. Nevertheless, the resistance genes’ transfer mechanisms are still poorly recognized, and this issue requires further investigations. Moreover, the relationship between operating conditions under which ARGs are developed or reduced should be also taken under consideration. Furthermore, the quantity of the ARGs should be analyzed in different depths of sand substrate in CWs.

The bacterial community in the CW columns exposed to SMX showed the ability to degrade it since four potential TPs-SMX were detected in the effluents. Among them, the conjugates of SMX with GSH were identified: hydroxy-glutathionyl-SMX and glutathionyl-SMX. This may suggest the presence of an oxidative stress in bacteria and plants and may confirm the participation of microorganisms in the transformation of SMX.

## Electronic supplementary material

ESM 1 (DOCX 851 kb)
