# Supplementary material for: The treatment of wastewater containing pharmaceuticals in microcosm constructed wetlands: the occurrence of integrons (int1–2) and associated resistance genes (sul1–3, qacEΔ1)
Source: Environ Sci Pollut Res Int. 2017 May 10;24(17):15055–66. doi: 10.1007/s11356-017-9079-1 (PMC5486623; doi:10.1007/s11356-017-9079-1)
Supplement: Supplementary file 1 — (DOCX 851 kb) [file 11356_2017_9079_MOESM1_ESM.docx]

**Supporting information for**

**Treatment of wastewater containing pharmaceuticals in microcosm constructed wetlands: the occurrence of integrons (*int*1-2) and associated resistance genes (*sul*1-3, *qac*EΔ1)**

**Monika Nowrotek^1,2*^, Ewa Kotlarska^3^, Aneta Łuczkiewicz^4^, Ewa Felis^1,2^, Adam Sochacki^1,2,5^, Korneliusz Miksch^1,2^**

^1^Silesian University of Technology, Environmental Biotechnology Department, ul. Akademicka 2, 44-100 Gliwice, Poland

^2^Silesian University of Technology, Centre for Biotechnology, ul. B. Krzywoustego 8, 44-100, Gliwice, Poland

^3^Institute of Oceanology Polish Academy of Sciences, Department of Genetics and Marine Biotechnology, ul. Powstanców Warszawy 55, 81-712 Sopot, Poland

^4^Gdansk University of Technology, Department of Water and Wastewater Technology, ul. Narutowicza 11/12, 80-233 Gdańsk, Poland

^5^Czech University of Life Sciences Prague, Faculty of Environmental Sciences, Department of Applied Ecology, Kamýcká 129, 165 00 Prague, Czech Republic

*Corresponding author: monika.nowrotek@polsl.pl


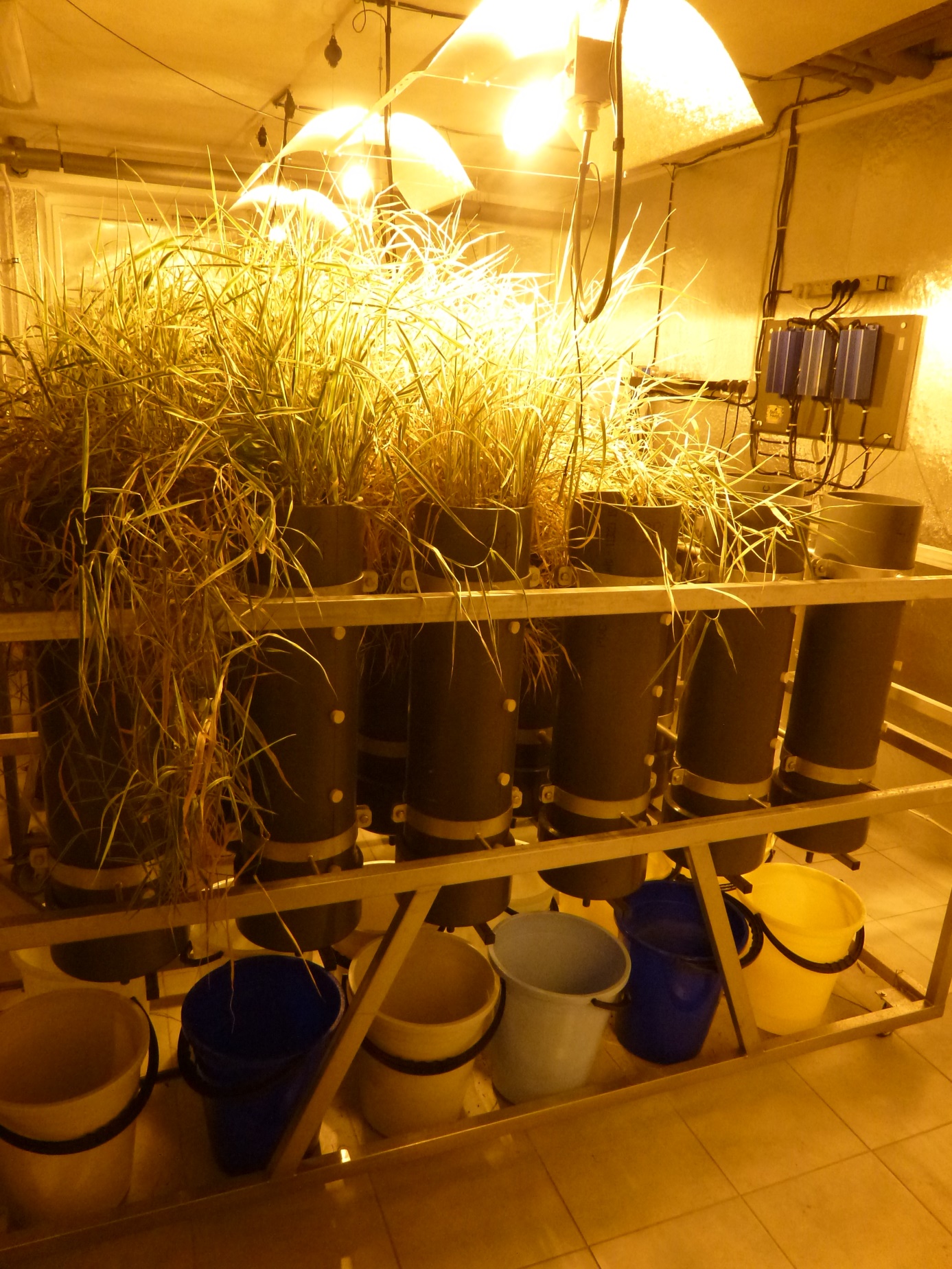


**Figure S1** Photo of the experimental system: a rig supporting 12 columns; the remaining 12 columns were mounted to an identical rig.

**Table S1**. The detailed information on the composition of the raw wastewater used in the study (based on Nowrotek et al. (2016)

| Component* | Concentration, mg/L | Concentration of metal cation, mg/L |
| --- | --- | --- |
| urea | 208.76 | ----- |
| NH_4_Cl | 62.45 | ----- |
| yeast extract | 264.00 | ----- |
| skim milk powder | 118.00 | ----- |
| sodium acetate | 510.40 | ----- |
| peptone | 40.00 | ----- |
| KH_2_PO_4_ | 41.37 | ----- |
| KCr(SO_4_)_2_∙12H_2_O | 0.96 | 0.100 |
| CuSO_4_∙5H_2_O | 0.781 | 0.200 |
| MnSO_4_∙H_2_O | 0.108 | 0.035 |
| NiSO_4_∙7H_2_O | 0.359 | 0.075 |
| PbCl_2_ | 0.100 | 0.075 |
| ZnCl_2_ | 0.208 | 0.100 |
| MgSO_4_∙7H_2_O | 4.408 | 0.430 |

*The influent was prepared in tap water by dissolving the following components

**Table S2.** Primers used in PCR experiments

| **Target** | **Primer sequences (5’-3’)** | **PCR product size (bp)** | **PCR annealing temperature (°C)** | **Reference** |
| --- | --- | --- | --- | --- |
| ***int1*** | int1AF: CCT CCC GCA CGATGATC  int1AR: TCC ACG CAT CGT CAG GC | 280 | 55 | Kraft et al. (1986) |
| ***int*2** | int2AF: TTATTG CTG GGATTA GGC  int2AR: ACG GCT ACC CTC TGT TAT C | 233 | 50 | Goldstein et al. (2001) |
| ***qac*EΔ1** | qacF: ATC GCA ATA GTT GGC GAA GT  qacR: CAA GCT TTT GCC CAT GAA GC | 225 | 55 | Stokes and Hall (1989) |
| ***sul1*** | sul1F: ATG GTG ACG GTG TTC GGC ATT CTG A  sul1R: CTA GGC ATG ATC TAA CCC TCG GTC T | 800 | 55 | Grape et al. (2003) |
| ***sul*2** | sul2-F GAATAAATCGCTCATCATTTTCGG  sul2-R CGAATTCTTGCGGTTTCTTTCAGC | 722 | 50 | Toleman et al. (2006) |
| ***sul*3** | sul3-F GAGCAAGATTTTTGGAATCG  sul3-R CATCTGCAGCTAACCTAGGGCTTTGGA | 792 | 51 | Perreten and Boerlin (2003) |

**Table S3.** The occurrence the analysed genes (*sul*1-3, *int*1-2 and *qac*EΔ1) in the column replicates at various stages of the CW experiment

| **Column** | **Replicate** | **PhCs in the feed** | | |
| --- | --- | --- | --- | --- |
|  |  | **Day 7** | **Day 22** | **Day 47** |
| **Upper layer of substrate** | | | | |
|  | I | *sul*2 | *sul*1, *sul*2, *int*1, *qac*EΔ1 | *sul*1 |
|  | II | *sul*1, *sul*2, *int*1 | *sul*1, *int*1, *qac*EΔ1 | *sul*1 |
| **PhCs-P** | III | *sul*2 | *sul*1, *sul*2, *int*1, *qac*EΔ1 | nd. |
|  | IV | nd. | *sul*1, *sul*2, *int*1, *qac*EΔ1 | *sul*1, *sul*2, *int*1 |
|  | V | *sul*1, *sul*2, *int*1 | *sul*1, *sul*2, *int*1, *qac*EΔ1 | *sul*1 |
|  | VI | nd. | *sul*1, *sul*2, *int*1, *qac*EΔ1 | *sul*1 |
|  | I | *sul*2 | nd. | nd. |
|  | II | *sul*2 | *sul*2 | nd. |
| **noPhCs-P** | III | *sul*2 | *sul*1, *sul*2, *int*1 | *sul*1 |
|  | IV | nd. | nd. | nd. |
|  | V | nd. | nd. | nd. |
|  | VI | *sul*1, *sul*2, *int*1 | *sul*1, *sul*2, *int*1 | *sul*1 |
|  | I | nd. | nd. | nd. |
|  | II | nd. | *sul*1, *sul*2, *int*1, *qac*EΔ1 | nd. |
| **PhCs-U** | III | nd. | *sul*1, *int*1, *qac*EΔ1 | nd. |
|  | IV | *sul*2, *int*1 | *sul*1, *sul*2, *int*1, *qac*EΔ1 | *sul*1 |
|  | V | nd. | *sul*1, *sul*2, *int*1, *qac*EΔ1 | *sul*1 |
|  | VI | *sul*1, *sul*2, *int*1 | *sul*1, *sul*2, *int*1, *qac*EΔ1 | *sul*1, *int*1 |
|  | I | nd. | nd. | nd. |
|  | II | nd. | nd. | nd. |
| **noPhCs-U** | III | nd. | nd. | nd. |
|  | IV | nd. | *sul*2 | nd. |
|  | V | nd. | nd. | nd. |
|  | VI | *sul*2, *int*1 | *sul*1 | nd. |
| **CW effluents** | | | | |
| **PhCs-P**  **effluent** | I | nt. | *sul*1, *sul*2, *int*1, *qac*EΔ1 | nt. |
|  | II | nt. | *sul*1, *sul*2, *int*1, *qac*EΔ1 | nt. |
|  | III | nt. | *sul*1, *sul*2, *int*1, *qac*EΔ1 | nt. |
|  | IV | nt. | *sul*1, *sul*2, *int*1, *qac*EΔ1 | nt. |
|  | V | nt. | *sul*1, *sul*2, *int*1, *qac*EΔ1 | nt. |
|  | VI | nt. | *sul*1, *sul*2, *int*1, *qac*EΔ1 | nt. |
| **PhCs-U**  **effluent** | I | nt. | *sul*1, *sul*2, *int*1, *qac*EΔ1 | nt. |
|  | II | nt. | *sul*1, *sul*2, *int*1, *qac*EΔ1 | nt. |
|  | III | nt. | *sul*1, *sul*2, *int*1, *qac*EΔ1 | nt. |
|  | IV | nt. | nd. | nt. |
|  | V | nt. | *sul*1, *int*1, *qac*EΔ1 | nt. |
|  | VI | nt. | *sul*1, *int*1, *qac*EΔ1 | nt. |

*^)^nd – not detected, **^)^nt – not tested

**References**

Goldstein C, Lee MD, Sanchez S, Hudson C, Philips B, Register B, Grady M, Liebert C, Summers AO, White DG, Maurer JJ (2001) Incidence of class 1 and 2 integrases in clinical and commensal bacteria from livestock, companion animals, and exotics. Antimicrob Agents Chemother 45:723–726. doi: 10.1128/AAC.45.3.723-726.2001

Grape M, Sundström L, Kronvall G (2003) Sulphonamide resistance gene *sul*3 found in *Escherichia coli* isolates from human sources. J Antimicrob Chemother 52:1022–1024. doi: 10.1093/jac/dkg473

Kraft CA, Timbury MC, Platt DJ (1986) Distribution and genetic location of Tn7 in trimethoprim-resistant *Escherichia coli*. J Med Microbiol 22(2):25–131.

Nowrotek M, Sochacki A, Felis E, Miksch K (2016) Removal of diclofenac and sulfamethoxazole from synthetic municipal waste water in microcosm downflow constructed wetlands: Start-up results. Int J Phytoremediation 18(2):157-163. doi: 10.1080/15226514.2015.1073669.

Perreten V, Boerlin P (2003) A new sulfonamide resistance gene (*sul*3) in *Escherichia coli* is widespread in the pig population of Switzerland. Antimicrob Agents Chemother 47(3):1169-1172.

Stokes HW, Hall RM (1989) A novel family of potentially mobile DNA elements encoding site-specific gene-integration functions: integrons. Mol Microbiol 3:1669–1683.

Toleman MA, Bennett PM, Walsh TR (2006) ISCR elements: novel gene-capturing systems of the 21st century? Microbiol Mol Biol Rev 70(2), 296-316.
